# Supplementary material for: How does fintech affect bank efficiency in Taiwan?
Source: PLoS One. 2023 Aug 7;18(8):e0289629. doi: 10.1371/journal.pone.0289629 (PMC10406230; doi:10.1371/journal.pone.0289629)
Supplement: Attachment — Submitted filename: Review report.docx [file pone.0289629.s003.docx]

**The Impact of Fintech on Bank Efficiency: Application of parametric and non-parametric approaches**

**Review Report**

I get the chance to read this paper. It is an interesting paper which aims to determine the relationship between bank efficiency and Fintech related-business. However, the author is unable to find any significant relationship.

**Introduction**

I am not satisfied with the introduction session. The current version of the introduction is emphasising more about literature and the importance of Fintech firms and Taiwan's economy:

1. The important part was the contribution of the study, which I was unable to find. I would encourage the author to look into the importance of this study. Like if this study is not conducted, what would happen?
2. The? manuscript is redundant as the literature mentioned in the introduction session is also cited in the literature review session. Hence, the introduction was unable to introduce the topic.
3. The introduction part needs to include the practical implications of the topic.

The Fintech operations are beneficent for the economy and the efficiency of banks. If the shareholder is holding a zero portfolio, where did he need to invest? Should he/she invest in the banking sector after the Fintech related-businesses are commenced or before? Also, what is the impact of Fintech businesses on the overall economy? Can it help strengthen the economy and pay back the debt of Taiwan, or to what extent?

There are several studies which discuss the impact of Fintech-related services on the performance and efficiency of the banking sector (please see references below). In the last, I failed to see the gap in my study. What is new about this study? The author does not justify the novelty of the study.

Given the above reason, this paper can improve the current version of the introduction.

**References:**

Chen, X., You, X., & Chang, V. (2021). FinTech and commercial banks' performance in China: A leap forward or survival of the fittest?. *Technological Forecasting and Social Change*, *166*, 120645.

Nastiti, N. D., & Kasri, R. A. (2019). The role of banking regulation in the development of Islamic banking financing in Indonesia. *International Journal of Islamic and Middle Eastern Finance and Management*.

Zhao, J., Li, X., Yu, C. H., Chen, S., & Lee, C. C. (2022). Riding the FinTech innovation wave: FinTech, patents and bank performance. *Journal of International Money and Finance*, *122*, 102552.

**Literature Review**

The paper's literature session is a weakness in terms of theoretical background. There is no grounding theory related to this topic mentioned by the author. There are several efficiency theories pertaining to the efficiency theory and bank performance. Without proper background theory, I am unable to see the relevance of the literature review.

The hypotheses development is poorly defined, such as the first hypothesis being more appropriate for the methodology session. The author needs to realise what is testing, whether this is a Fintech-related service on the bank efficiency or a new methodology introduced by him.

I am concerned by the proxies of the Fintech related-business. The author mentioned, "Generally, fintech refers to the combination of finance and information technology, using IT-related activities to improve bank efficiencies, such as ATM, credit card, internet banking, and mobile payment.". The proxies for Fintech taken is from industry 3.0, where ATMs are considered a big development in the banking infrastructure. We are standing at Industrial revolution 4.0 and moving to industrial revolution 5.0. These proxies for Fintech-related business can not be called "Fintech" in our study now. I agree that those were significant developments around two decades back, but do these proxies have any relevance in today's world? This question is important to understand as the whole foundation of this paper depends on the proxied for the Fintech related-business. In addition, mobile payments have a positive impact on bank efficiency. Indeed, there are shreds of evidence already researched the Fintech involvement in the banking sector. The author did not emphasise what is new about this paper.

The 4th hypothesis the author mentioned "*It is also a critical measure of the effect of Fintech is the bank's IT investment. To what extent have IT investments impacted firm efficiency and productivity? While studies argue that IT investments can allow firms to achieve both revenue growth and cost savings (Mithas et al. 2012), Strassman (2002) insists that IT investment cannot contribute to productivity or corporate performance as expected. Investment in IT has grown remarkably; however, the growth of productivity in individual firms and in the entire economy has been relatively slow. Beccalli (2007) found little relationship between IT investment and improved bank profitability or efficiency, indicating the existence of a profitability paradox. Gupta et al. (2018) confirm the profitability paradox in the Indian banking sector. This phenomenon raises doubts regarding IT's ability to fulfil benefits that once seemed promising (Rao and Mandal 2012). Ou et al.(2009) found that IT investment is associated with positive bank efficiency in Taiwan. However, numerous studies cannot appropriately explain the performance resulting from IT investments*"

The references are from 2012 (one decade back) and more; several studies were conducted during the last decade on this topic. Considering expenditure on IT equipment is not an appropriate proxy for the Fintech firms. The firms may buy new computers rather than a cybersecurity system (Fintech). I would recommend removing this hypothesis better to understand the Fintech businesses with the efficiency of banks.

**Methodology:**

The authors do a good job in this session. They ascertain the efficiency of the bank by using the DFA, SAF, and DEA approaches. Then, they run the t-test and one-way ANOVA for all these three models. Afterwards, the author used these proxies for the regression models and saw the impact of Fintech services (proxied as mentioned above) on the efficiency of banks. However, the regression models (Table 9) do not control for other bank-level factors, such as leverage or ownership structure.

For the robustness test, the authors should use an alternative proxy for the Fintech firms and efficiency. Such as, there are several efficiency ratios at the bank level. I suggest reading the recent paper by Dhiaf et al. (2022). The paper examines the impact of Fintech firms on manufacturing efficiency. I recommend to the author that he can take similar proxies for the robustness test.

**Reference**:

Dhiaf, M. M., Khakan, N., Atayah, O. F., Marashdeh, H., & El Khoury, R. (2022). The role of FinTech for manufacturing efficiency and financial performance: in the era of industry 4.0. *Journal of Decision Systems*, 1-22.

**Conclusion:**

The conclusion session is up to a satisfactory level, but it needs the future studies part of the conclusion, like how future studies can improve the current version of this paper and can contribute to it.
