# Supplementary material for: How does fintech affect bank efficiency in Taiwan?
Source: PLoS One. 2023 Aug 7;18(8):e0289629. doi: 10.1371/journal.pone.0289629 (PMC10406230; doi:10.1371/journal.pone.0289629)
Supplement: Attachment — Submitted filename: Response to Reviewers PONE-D-22-27977.docx [file pone.0289629.s004.docx]

Dear Editor:

Thank you for your valuable suggestions.

I also appreciate the time and effort you and each reviewer have dedicated to providing insightful feedback on ways to strengthen my paper. Thus, it is with great pleasure that we resubmit our article for further consideration. I have incorporated changes that reflect the detailed suggestions you have graciously provided. In order to make the research topic of this study easier to understand and focus it. I change the manuscript title to the new title: **How Does Fintech Affect Bank Efficiency in Taiwan?**

**Revision Notes:**

Thank two anonymous referees for their valuable suggestions and problems. The author responds to their suggestion in order as follows:

**Reviewer 1 Comments**

To facilitate your review of our revisions, the following is a point-by-point response to the questions and comments delivered in your letter. My main revised are as follows:

Thank you for this suggestion. These suggestions are replied to as follows:

**Introduction**

Thank you for this suggestion. These suggestions are replied to as follows:

First, the “introduction” was major revised. I attempted to make this article describe the impact of fintech on the banking industry more clearly. For example, Arner et al. (2016) and Chen et al. (2021), argued that the Fintech revolution has three phases. This study defined the Fintech revolution process to classify three-stage, Fintech 1.0 (before 1967), Fintech 2.0 (1967-2008) and Fintech 3.0 (after 2008), respectively. Please check Chapter “Introduction” on pages 2-3.

Second, more detail describes the contribution of this study on page 3.

The contribution of this study is explained in the following. First, few studies have considered fintech-related initiatives in the context of bank efficiency; therefore, this is the first study to consider fintech-related initiatives in connection to bank efficiency. Second, to the best of my knowledge, this study is the first to explain the different impacts of Fintech 2.0 and Fintech 3.0 on bank efficiency. Financial products and services related to Fintech 2.0 include ATMs and telephone banking. Financial products and services related to Fintech 3.0 include mobile payment and blockchain services. Third, this study employs parametric and non-parametric approaches to evaluate bank efficiency. Fourth, existing literature often used macroeconomic-level (firm-level) to investigate the relationship between Fintech and economic growth (risk-taking). This study uses bank-level data to examine the effect of fintech on the banking industry. Fifth, Taiwan has a complete IT infrastructure, talent pool, and industrial chain. In sum, this study aims to answer the question: can banks improve their efficiency through fintech?

**Literature Review**

Thank you for this suggestion. This study evaluates bank efficiency by considering the fintech-related initiative as an output variable in cost function modelling. I re-write the first hypothesis as below:

$H_{1}$***:*** *Fintech-related initiative affect bank efficiency.*

**Data and methodology**

**Data**

Thank you for this suggestion. I add the proper descriptive statistics of variables, such as Max. and Min. Please check Table 1.

**Model specification**

Thank you for this suggestion. These suggestions are replied to as follows:

First, following Bauer et al. (1998), two outputs are total loans ($Y_{1}$) and total deposits ($Y_{2}$), which are commonly used in the literature. Avoiding the revenue fee as output while using the transaction value of ATMs and credit cards as fintech-related initiatives outputs maybe cause a biased outcome.

Second, following Berger et al. (2005), this study uses selection indicator dummy variables. MPAY is a dummy variable whose value is 1 if the bank cooperated with Taiwan Mobile Payment Co. Ltd. (TWMP, a mobile payment systems developer since 2014) and 0 otherwise; TPAY is a dummy variable whose value is 1 if the bank used TWMP’s payment app system and 0 otherwise.

MPAY as a dummy variable in each dummy variable equal to zero before the bank did not cooperate with TWMP; when the bank started the year's collaboration with TWMP dummy variable was equal to one. Furthermore, customers used TWMP’s payment system to make deals, pay taxes, and transfer money. It is a tokenization payment platform for financial institutions that began in 2017. Thus, TPAY is a dummy variable whose value is 1 if the bank used TWMP’s payment service and 0 otherwise.

Third, this typo has been corrected in the chapter “**Methodology**”.

**Empirical Results**

Thank you for this suggestion.

First, definitions of external regressors have been provided in the notes to each table.

Second, I tested different robustness checks based on different estimation methods, such as two-stage least squares (2SLS). This study also tested the alternative definitions of the proxy variables for Fintech 2.0 and Fintech 3.0 (e.g. blockchain correspondence service (BLC), number of Taiwan-pay users (TCD) and amounts of transactions of Taiwan-pay (TAM)) and bank performance (e.g., ROA) to address the reliability of my findings.

**CONCLUDING REMARKS**: Again, thank you for giving me the opportunity to strengthen my manuscript with your valuable comments and queries. I have worked hard to incorporate your feedback and hope that these revisions persuade you to accept this submission.

**References**

1. Arner, D.W., Barberis, J.N. and Buckley, R.P., The Evolution of Fintech: A New Post-Crisis Paradigm? *Georgetown. Journal International Law*, **2016**, 47, 1271.
2. Bauer, W. Berger, N. Ferrier, D. and Humphrey, B., Consistency conditions for regulatory analysis of financial institutions: a comparison of frontier efficiency methods, *Journal of Economics and Business*, **1998**,50, 85-114. https://doi.org/10.1016/S0148-6195(97)00072-6
3. Berger, A.N., Clarke, G., Cull, R., Klapper, L. and Udell, G.F., Corporate governance and bank performance: A joint analysis of the static, selection, and dynamic effects of domestic, foreign, and state ownership, *Journal of Banking and Finance*, **2005**, 29, 2179-2221. <https://doi.org/10.1016/j.jbankfin.2005.03.013>
4. Chen, X., You, X. and Chang, V., FinTech and commercial banks' performance in China: A leap forward or survival of the fittest?, *Technological Forecasting and Social Change*, **2021**,166,120645. https://doi.org/10.1016/j.techfore.2021.120645.

**Reviewer 2 Comments**

To facilitate your review of our revisions, the following is a point-by-point response to the questions and comments delivered in your letter. My main revised are as follows:

**Introduction**

Thank you for this suggestion. These suggestions are replied to as follows:

First, the “introduction” was major revised. Please check this chapter on page.2-4. I deleted some literature content and attempted to make this article describe the impact of fintech on the banking industry more clearly.

Second, following Arner et al. (2016) and Chen et al. (2021), they argued that the Fintech revolution has three phases. This study defined the Fintech revolution process to classify three-stage, Fintech 1.0 (before 1967), Fintech 2.0(1967-2008) and Fintech 3.0 (after 2008), respectively. For more detail describe, please see page 2.

Third, more detail describes the contribution of this study on page 3.

The contribution of this study is explained in the following. First, few studies have considered fintech-related initiatives in the context of bank efficiency; therefore, this is the first study to consider fintech-related initiatives in connection to bank efficiency. Second, to the best of my knowledge, this study is the first to explain the different impacts of Fintech 2.0 and Fintech 3.0 on bank efficiency. Financial products and services related to Fintech 2.0 include ATMs and telephone banking. Financial products and services related to Fintech 3.0 include mobile payment and blockchain services. Third, this study employs parametric and non-parametric approaches to evaluate bank efficiency. Fourth, existing literature often used macroeconomic-level (firm-level) to investigate the relationship between Fintech and economic growth (risk-taking). This study uses bank-level data to examine the effect of fintech on the banking industry. Fifth, Taiwan has a complete IT infrastructure, talent pool, and industrial chain. In sum, this study aims to answer the question: can banks improve their efficiency through fintech?

**Literature Review**

Thank you for this suggestion. These suggestions are replied to as follows:

First, the fintech revolution is fast and unpredictable. Finding reasonable variables representing Fintech has always been difficult in the existing literature. Due to unavailable data, thus, this study used alternative variables as a proxy for fintech, such as ATMs, credit cards, internet banking and mobile payment. To further, this study adds financial blockchain correspondence service as a Fintech 3.0 service and follows Banna et al. (2021); thus, I defined the blockchain correspondence service as a new type of service in the age of Industry 4.0. Thus, I also investigated the impact of post-industrial revolution eras (IR 4.0) on bank efficiency (since 2016).

Second, I rewrite hypothesis $H_{4}$, IT investment is not an appropriate proxy for the Fintech. Thus, I re-define and more clearly the 4^th^ hypothesis as below.

“IT investment is the core of fintech development. Thus, it indirectly affects bank efficiency. In this study, we answer the question: to what extent does IT investment impact bank efficiency? While studies have argued that IT investments increase revenue and savings (Mithas et al., 2012), Strassman (2002) insisted that it cannot contribute to productivity or corporate performance as expected. It is argued that IT investment has grown remarkably; however, the productivity of firms and the entire economy has been relatively slow. Beccalli (2007) found little to no connection between IT investment and bank efficiency, a “profitability paradox.” Gupta et al. (2018) confirmed this profitability paradox in the Indian banking sector. This phenomenon raises doubts regarding IT investments’ positive effects (Rao and Mandal, 2012). On the contrary, Ou et al. (2009) found that IT investment is positively associated with bank efficiency in Taiwan. Regardless, what is clear is that quantitative and qualitative studies on the relationship between IT investment and bank efficiency are sparse. Therefore, this study proposes the following hypotheses:

$H_{4a}$***:*** *IT investment has a positive impact on bank efficiency.*

$H_{4b}$***:*** *IT investment has a negative impact on bank efficiency.*

**Methodology**

Thank you for this suggestion. These suggestions are replied to as follows:

First, this study adds control variables for bank-level factors, such as EA indicates equity-assets ratio, and MANAGE indicates total operating revenue-total operating cost.

Second, I tested different robustness checks based on different estimation methods, such as two-stage least squares (2SLS). This study also tested the alternative definitions of the proxy variables for Fintech 2.0 and Fintech 3.0 (e.g. blockchain correspondence service (BLC), number of Taiwan-pay users (TCD) and amounts of transactions of Taiwan-pay (TAM)) and bank performance (e.g., ROA) to address the reliability of my findings.

**Conclusion**

Thank you for this suggestion. To add the description of future research as below: “Future research may examine these aspects in the context of the Greater China marketplace or emerging Asian economies to shed further light on the productivity paradox.”

**CONCLUDING REMARKS**: Again, thank you for giving me the opportunity to strengthen my manuscript with your valuable comments and queries. I have worked hard to incorporate your feedback and hope that these revisions persuade you to accept this submission.

**References**

1. Arner, D.W., Barberis, J.N. and Buckley, R.P., The Evolution of Fintech: A New Post-Crisis Paradigm? *Georgetown. Journal International Law*, **2016**, 47, 1271.

2. Banna, H., Hassan, M.K., and Rashid, M., Fintech-based financial inclusion and bank risk-taking: Evidence from OIC countries, *Journal of International Financial Markets, Institutions and Money*, **2021**, 75, 2021,101447. https://doi.org/10.1016/j.intfin.2021.101447.

3.Bauer, W. Berger, N. Ferrier, D. and Humphrey, B., Consistency conditions for regulatory analysis of financial institutions: a comparison of frontier efficiency methods, *Journal of Economics and Business*, **1998**,50, 85-114. https://doi.org/10.1016/S0148-6195(97)00072-6

4.Berger, A.N., Clarke, G., Cull, R., Klapper, L. and Udell, G.F., Corporate governance and bank performance: A joint analysis of the static, selection, and dynamic effects of domestic, foreign, and state ownership, *Journal of Banking and Finance*, **2005**, 29, 2179-2221. <https://doi.org/10.1016/j.jbankfin.2005.03.013>

5.Chen, X., You, X. and Chang, V., FinTech and commercial banks' performance in China: A leap forward or survival of the fittest?, *Technological Forecasting and Social Change*, **2021**,166,120645. https://doi.org/10.1016/j.techfore.2021.120645.

I hope to hear from you soon.

Thanking you in anticipation,

Sincerely,

Chang-Sheng Liao

School of Economics and Management, Hubei Polytechnic University, Hubei, China

Department of Finance and Cooperative Management, National Taipei University, Taiwan, Republic of China
