# Supplementary material for: How does fintech affect bank efficiency in Taiwan?
Source: PLoS One. 2023 Aug 7;18(8):e0289629. doi: 10.1371/journal.pone.0289629 (PMC10406230; doi:10.1371/journal.pone.0289629)
Supplement: Attachment — Submitted filename: Response to Reviewers PONE-D-22-27977 R2.docx [file pone.0289629.s005.docx]

Dear Editor:

Thank you for your valuable suggestions.

I also appreciate the time and effort you and each reviewer have dedicated to providing insightful feedback on ways to strengthen my paper. Thus, it is with great pleasure that we resubmit our article for further consideration. I have incorporated changes that reflect the detailed suggestions you have graciously provided.

**Revision Notes:**

Thank two anonymous referees for their valuable suggestions and problems. The author responds to their suggestion in order as follows:

**Reviewer 1 Comments**

To facilitate your review of our revisions, the following is a point-by-point response to the questions and comments delivered in your letter. My main revised are as follows:

Thank you for this suggestion. These suggestions are replied to as follows:

**Introduction**

Integrating the referee's suggestions, the author made the following revisions. First, the author moves background information on the fintech evolution in Taiwan in the section” Background”. Please check on page 3.

Second, the authors add to describe the IT Productivity Paradox on page 16. “*The IT Productivity Paradox (or the Solow paradox) indicates that productivity growth may not increase despite sizeable investment in IT (Shu and Strassmann, 2005)*”.

Third, the author explains that the existing literature has some weak, and this study will address the gap. *Understanding the impact of IT (Fintech) on bank efficiency is important because it incurs high costs and influences bank operations and strategies (Beccalli, 2007) ……. However, these studies do not consider Fintech evolution or the different stages in Fintech that affect bank efficiency. Thus, here we provide an interesting case study on the effects of Fintech initiatives, such as ATMs (Fintech 2.0), mobile payment (Fintech 3.0), and IT investment.* Please check page 2 for details.

Fourth, the author re-wrote the section "contribution" and tried to explain this study and provide some highlights compared to previous literature.

**Literature Review**

Integrating the referee's suggestions, the author made the following revisions.

First, this manuscript has been to re-English edited by EDITAGE. Please see attached file for certificate documents.

Second, the author has minor revised H2 and added some describe why to separate these into distinct hypotheses based on different conditions.

*Humphrey and Berger (1990) analyzed payment system costs. Their findings showed that cash was the cheapest payment system from a social cost perspective, followed by point-of-sale and ATM bill payments. Frame and White (2004) indicated that financial innovation could refer to new services, improved production processes, or organizational forms of innovation. This finding implies that Fintech positively impacts firm performance. However, Cook et al. (2004) highlighted the importance of increasing service delivery speed and significantly decreasing costs through ATMs, telephone banking, and other online and electronic means. Sathye and Sathye (2017) found that ATM intensity had a significant negative association with bank efficiency, and ascribed this to banks’ investment in Fintech—such as ATMs—and their inability to reduce labor costs, given that many processes continue to be manual. Kondo (2010) found that ATMs did not influence banks’ return on assets (ROA) in Japan. Fintech provides a variety of new channels, such as internet banking, telephone banking, and ATMs. It contributes to the precision, speed, and confidentiality of customer transactions, while boosting customer convenience (Liao,2022). However, empirical results are mixed regarding the relationship between ATMs and bank efficiency. Based on these arguments, this study proposes the following hypotheses:*

Third, the author has added some empirical studies to strengthen the proposed hypotheses. Please see the section “Literature reviews”.

Fourth, I think your suggestion is good. Resource-Based View (RBV) or the Dynamic Capabilities View (DCV) helps explain IT capabilities can contribute to firm efficiency. However, we have carefully evaluated the funding and resources required to complete these additional studies and found that such an expanded study is currently out of stock. Meanwhile, the scope of work of the present paper can support its conclusions. Therefore, I suggest the additional work be included in a follow-up paper.”

**Methodology**

Thank you for this suggestion. These suggestions are replied to as follows:

That is a very good question. I apologize for may not express myself clearly. The results of VIF some studies use a single table; for example, Nenavath and Mishra (2023). As Like below in Table 1. The author considers conserving space; thus, please agree me only insert VIF results into Table 5.

Table 1 Detecting multicollinearity using VIF.

| Variables | VIF | 1/VIF |
| --- | --- | --- |
| ***ATM*** | **3.328** | 0.3005 |
| ***ITE*** | **1.076** | 0.9294 |
| ***MPAY*** | **1.31** | 0.7634 |
| ***TPAY*** | **1.442** | 0.6935 |
| ***SIZE*** | **3.944** | 0.2535 |
| ***NPL*** | **1.307** | 0.7651 |
| ***EA*** | **1.203** | 0.8313 |
| ***MANAGE*** | **1.426** | 0.,7013 |

**Conclusion**

Thank you for this suggestion. These suggestions are replied to as follows:

First, the author added the “limitation of the study” in the Conclusion. *This study also has some limitations, primarily owing to limitations on data accessibility, making analysis difficult, and ultimately impacting the comprehensiveness of the study model. Given the data availability issues, this study used a select dummy variable model and mobile payment data as proxy Fintech 3.0. However, to accurately quantify Fintech 3.0 (further, Fintech 4.0) additional discussion is needed.* Please check on page 24.

Second, the author more detail describes “Future Research” in the Conclusion. *Academic research is progressive, making it important to gather pertinent data and conduct further studies on how Fintech affects bank efficiency (performance). Future research may examine these aspects in the context of the Greater China region or emerging Asian economies to untangle the productivity paradox. Especially, Fintech has made significant progress in the Chinese financial market, but whether Fintech has already played a role in China's banking industry remains to be further studied. It is suggested that future research consider text mining, and weight indicators by the entropy method and grey relational analysis to measure Fintech development levels.*

Please check on page 24.

Third, the author more detail describes “Practical Implications” in the Conclusion; *This study has several policy implications. First, from a policy perspective, the government should provide more tax advantages, encourage banks to enlarge IT investment, and allow more non-banking Fintech enterprises to enter the financial market. This would benefit Fintech development and indirectly improve bank efficiency. However, appropriate policy initiatives (e.g., privacy and intellectual property laws) need to be considered. Second, from a managerial perspective, banks investing heavily in Fintech do not necessarily have efficient production processes. They may differ from efficient banks in ways that cannot be rectified by merely increasing their IT investments. Thus, excellent management is crucial. Managers should focus on improving Fintech planning, design, delivery, and strategies.* *Furthermore, managers should pay attention to the Fintech input–output relationship: achieving the most significant amount of practical work using as little IT investment as possible.*

Please check on page 24.

Fourth, the author more detail describes the “Synthesis of Hypotheses” in the Conclusion; *The proposed model measured bank efficiency with fintech-related initiatives. The basic model measured bank efficiency without Fintech-related initiatives. Bank efficiency was significantly higher in the proposed model, supporting* $H_{1}.$*Therefore, fintech-related initiatives are determinants of bank efficiency; it positively impacts bank efficiency. These results show that initiatives related to Fintech 2.0 do not have a positive effect on bank efficiency in Taiwan (supporting* $H_{2b}$*). However, the initiatives related to Fintech 3.0 slightly improve bank efficiency (weakly support* $H_{3a}$*, but strongly reject* $H_{3b}$*). Finally, IT investments can increase bank efficiency, but their contribution is negligible (weak support* $H_{4a}$*). In summary, Fintech 3.0 improves bank efficiency significantly more than Fintech 2.0, and the efficiency of bank IT investment still needs improvement.* Please check on page 24.

**References**

1. Beccalli E, Does IT investment improve bank performance? Evidence from Europe, *Journal of Banking and Finance*, 2007; 31(7): 2205-2230. https://doi.org/10.1016/j.jbankfin.2006.10.022
2. Frame, W., Scott, and Lawrence J. White. Empirical Studies of Financial Innovation: Lots of Talk, Little Action? *Journal of Economic Literature*, 2004, 42 (1): 116-144.DOI: 10.1257/002205104773558065
3. Humphrey D, and Berger A, Market failure and resource use: economic incentives to use different payment instruments, in Humphrey D.B.(eds) The U.S payment system: efficiency, risk and the role of the Federal reserve, 1990, Springer, Dordrecht.
4. Kondo K, DO ATMs influence bank profitability in Japan? *Applied Economics Letters*, 2010; 17(3): 297-303. DOI: 10.1080/13504850701720379
5. Liao C.S, Do E-business Services Enhance Bank efficiency in Taiwan? *International Journal of information systems in the Service Sector* 2022;14(1):1-17. DOI: 10.4018/IJISSS.287575
6. Nenavath, S. and Mishra, S. (2023). Impact of green finance and fintech on sustainable economic growth: Empirical evidence from India, *Heliyon*, 9(5), e16301, https://doi.org/10.1016/j.heliyon.2023.e16301.
7. Sathye S, and Sathye M, Do ATMs increase technical efficiency of banks in a developing country? Evidence from Indian banks, *Australian Accounting Review*, 2017;27(1): 101-111. https://doi.org/10.1111/auar.12110
8. Shu W, and Strassmann P.A, Does information technology provide banks with profit? *Information and Management*, 2005; 42: 781-787. https://doi.org/10.1016/j.im.2003.06.007

**CONCLUDING REMARKS**: Again, thank you for giving me the opportunity to strengthen my manuscript with your valuable comments and queries. I have worked hard to incorporate your feedback and hope that these revisions persuade you to accept this submission.

I hope to hear from you soon.

Thanking you in anticipation,

Sincerely,

Chang-Sheng Liao

School of Economics and Management, Hubei Polytechnic University, Hubei, China

Department of Finance and Cooperative Management, National Taipei University, Taiwan, Republic of China
