# Supplementary material for: How does fintech affect bank efficiency in Taiwan?
Source: PLoS One. 2023 Aug 7;18(8):e0289629. doi: 10.1371/journal.pone.0289629 (PMC10406230; doi:10.1371/journal.pone.0289629)
Supplement: Attachment — Submitted filename: PlosOne reviewer comments.docx [file pone.0289629.s002.docx]

The Impact of Fintech on Bank Efficiency: Application of parametric and non-parametric approaches

**Journal:** PLOS ONE

**Summary**

This study investigates the determinants of bank cost efficiency in Taiwan.

After reading this paper draft, I would like to raise some major concerns for further action at your desk.

**Introduction**

The contribution of this study is not clearly explained.

More especially, the word “fintech” seems to be overused and misinterpreted in this manuscript unless a convincing and clear definition of fintech is presented. Therefore, this study tends to examine the impact of delivery channels such as ATMs, and bank cards on bank efficiency. Please see Le and Ngo (2020) for a more comprehensive discussion.

**Literature review**

The first hypothesis is not really correct. Please rewrite it properly.

**Data and methodology**

**Data**

**Table 1**: Please provide a brief description of the variables used. Also please provide proper descriptive statistics of variables (e.g., min, max,..).

There are missing descriptive statistics for some important regressors.

**Model specification**

I am not convinced that the transaction value of ATMs and credit cards is used as two additional outputs. When these delivery services increase, banks would earn more fees. Therefore, the second output (revenue fee) has already accounted for this perspective. That is why I am not surprised that the results of efficiency scores are insignificant different when these two additional outputs are included/excluded.

Please reconsider this suggestion in your revision.

In equation (8), there are a few issues here:

- EPAY or MPAY? This variable should be measured by the transaction value of the mobile payment service. Unfortunately, the dummy variable is used. However, there is a disadvantage to using this. For example, can a firm change the status of offering mobile payment service over time (e.g., static indicator, selection indicator..)? Please see Berger et al. (2005). It would be same as the case of TELEP.
- I am very confused about the definitions of regressors. Please keep them consistency.
- Non-performing loans?

**Results**

**Regression analysis**

Please provide definitions of external regressors in the note in each table.

I am not going to make further comments on this section unless these above issues are well explained. If the contribution of this study is not well articulated, the rest of this paper will look like to be a data-crunching exercise in the context of the Taiwanese market.

**References**

Berger, Allen N, George RG Clarke, Robert Cull, Leora Klapper and Gregory F Udell (2005), Corporate governance and bank performance: A joint analysis of the static, selection, and dynamic effects of domestic, foreign, and state ownership, *Journal of Banking & Finance,* 29**,** 2179-2221.

Le, Tu DQ and Thanh Ngo (2020), The determinants of bank profitability: A cross-country analysis, *Central Bank Review,* 20**,** 65-73.
